# Qualitative Study of Inpatient Hospital Medicine Interruptive Alerts and Clinical Decision Support

**DOI:** 10.1055/a-2955-9555

**Published:** 2026-10-12

**Authors:** Afifah Khan, Jeffrey Schnipper

**Affiliations:** 1 Department of General Internal Medicine 370908 Brigham and Women’s HospitalBostonMassachusettsUnited States; 2 1811 Harvard Medical SchoolBostonMassachusettsUnited States

**Keywords:** clinical information systems, specific types, process management tools, clinical decision support, alerting, alert fatigue, clinical care, environment, inpatient care, patient records, electronic health records and systems, socio-technical aspects of information technology, people, governance

## Abstract

**Background** Although interruptive alerts, or pop-ups, are one of the most common forms of clinical decision support (CDS) tools within the electronic health record (EHR), they are often ignored. Interruptive alerts have the potential to enhance patient care and reduce clinician burden, but end-users frequently experience alert fatigue and override alerts, defeating their purpose. Few studies have elicited the perspectives of all stakeholder groups involved in the development, experience, and review of inpatient interruptive alerts in the modern vendor EHR era.

**Objectives** This study aimed to understand the perspectives of multiple stakeholders involved in development, governance, and use of inpatient interruptive alerts.

**Methods** We conducted a qualitative study, interviewing 22 participants, including 15 end-users, 4 governance group members, 2 clinical leads, and 1 information systems staff member. The semi-structured interview guides were designed to explore different participant perspectives on interruptive alerts.

**Results** We found three domains across end-users related to interruptive alert helpfulness, proportionality, and alarm fatigue. We found three domains across the governance, clinical lead, and information system groups about the causes of high-volume and high-sensitivity interruptive alerts, differing definitions of interruptive alert success from end-users, and reluctance to add new interruptive alerts but limited changes or retirement of existing interruptive alerts. Barriers to successful CDS could be grouped into end-user, technical, and governance-level barriers. We found one governance-level facilitator and gathered several recommendations for CDS improvement from all stakeholder groups, including changing alert “interruptiveness,” customizability, design, responsiveness, and involving end-users in alert analysis.

**Conclusion** This study highlights the need for robust, user-centered alerts and CDS development and evaluation to improve clinician experiences and potentially patient outcomes.

## Background and Significance

Clinical decision support (CDS) within the electronic health record (EHR) has the potential to improve the efficiency and safety of patient care by providing clinicians with information to make the best patient care decisions. 1 2 3 Patient data reports (e.g., dashboards), order sets, clinical workflow tools, computerized guidelines, and documentation templates have been designed with the intention of helping clinicians take better care of patients. 4 Interruptive alerts, or pop ups that require end-user action or acknowledgment, are one of the dominant forms of CDS and have the potential to distract from patient care and increase clinician’s cognitive load. 5 6 7 8 9 10

Although it is difficult to quantify interruptive alert burden, they are a commonly used form of CDS, and previous research has shown negative unintended consequences for end-users. 1 11 12 One study showed that critical care patients generate approximately 100 EHR alerts for end-users per day. 13 Another study showed that at a large academic healthcare system, interruptive alerts fired 53,106 times per month in the inpatient setting, representing 73.6% of all alerts fired in the system and was equivalent to approximately 71 hours of total provider effort per month. 14 Optimal alert volume is unknown, but alerts fire so frequently that most are ignored, with prior research demonstrating rates of ignoring as high as 96%, almost entirely defeating their purpose. 5 15 16 17 18 19 Alternative forms of CDS may result in decreased cognitive load and decreased patient harm. 20 21 In addition, more judicious use of interruptive alerts may lead to a higher rate of meaningful alerts noticed and accepted. 9 11 Finally, alert creation and maintenance can be quite costly. One study found the cost of creating 94 new drug alerts to be $23,694 to 48,668 (United States dollars), or approximately $252 to 518 per alert, with an additional annual maintenance cost of 10–20% of the creation cost. 22

Although Osheroff’s Five Rights of CDS is commonly acknowledged as the backbone of effective CDS, CDS broadly does not consistently employ the five rights, which likely contributes to CDS falling short of its potential. 23 Previous qualitative studies to better understand stakeholder perspectives on inpatient CDS and interruptive alerts have been limited. Bell et al demonstrated optimizing recipient targeting, timing, and alert design may minimize alert overrides in the inpatient cardiology setting. 24 Few, if any, recent studies have focused directly on the cumulative effects of inpatient interruptive alerts on end-users and the perspectives of other stakeholders involved in the development, experience, and review of alerts, especially in the current era where a small number of vendor EHRs dominate the inpatient setting. 25

## Objectives

The goal of this study was to better understand the perspectives of multiple stakeholders involved in development, governance, and usage of inpatient interruptive alerts and CDS.

## Methods

We conducted a qualitative study using phone or video interviews with 22 participants, 10 female and 12 male, at Brigham and Women’s Hospital, part of Mass General Brigham, a large integrated healthcare system in Eastern Massachusetts. The Consolidated Criteria for REporting Qualitative (COREQ) research guidelines were followed. 26 We interviewed stakeholders from each step of development, experience, and review of an interruptive alert to capture various perspectives on alerts, including responding clinicians, governance group members, site-specific clinical leaders, and information systems leaders. Responding clinicians are inpatient clinicians who write orders during hospitalization and are recipients of inpatient alerts, i.e., who could provide end-user perspectives. Governance groups are members of specialty committees (e.g., nursing, medication safety) with various backgrounds who make decisions about requests for new interruptive alerts and receive requests from the information systems team for interruptive alert modification or retirement. Clinical leads review requests for new CDS from their hospital site and work with governance groups and information systems to design CDS. Finally, the information systems team is a system-wide group of clinical and technical leads who create and maintain CDS within the EHR. Factors that lead to new interruptive alert requests, that go into the decision to create an alert, and that lead to best practice advisories (BPAs) being re-evaluated are shown in in **Supplementary Table S1** ( **Supplementary Appendix** ; available in the online version only).

Approximately 444 responding clinicians (physicians and advanced practice providers from the hospital medicine unit, internal medicine residents, and residents of other specialties rotating on internal medicine clinical services) were initially contacted by email via distribution lists, with four positive responses. Eleven responding clinicians were specifically identified by other responding clinicians as possibly having an interest in participating and responded positively to personalized emails. Nine people from the other stakeholder groups were identified via purposive sampling by experts within the healthcare system and emailed individually, with seven positive responses. All responding participants consented to participate, no participants dropped out, and approximately 431 participants from all stakeholder groups declined to participate by not responding to the email requests. Interviews were 10–30 min in length, and no repeat interviews were conducted. A semi-structured interview guide was created specific to each stakeholder group, using domains from prior conversations with institutional experts in CDS. The interview guide was not provided to participants ahead of time.

Individual interviews were conducted by Dr. Afifah Khan between September 2023 and March 2024, during which she took field notes. All interviews were audio-recorded, transcribed, de-identified, and transferred to DeDoose (SocioCultural Research Consultants, Los Angeles, California, United States) for data analysis. Interviews were conducted virtually at home or in the workplace with no other people present besides the participants and the researcher. The initial codes for qualitative domains and themes were created by both authors using conceptual frameworks from Osheroff’s Five Rights of Clinical Decision Support as well as broader principles of human-centered design. 27 Thematic and content analysis was conducted using the constant comparative method and a combination of inductive and deductive approaches. The two reviewers, Drs. Khan and Schnipper, iteratively revised codes from 22 interviews, independently coded data and met to resolve discrepancies, and created and refined themes and modified interpretations in an iterative fashion. Interviews were stopped when reviewers reached thematic sufficiency. 28 Transcripts were not returned to participants for review, and participants did not provide feedback on study findings. The coding tree is available upon request.

## Results

Participants included 15 responding clinicians from the Brigham and Women’s hospital medicine service, including 9 internal medicine residents, 5 advanced practice clinicians, and 1 attending hospitalist physician. We interviewed four governance group members, two of whom were pharmacists and two were nurses. We also interviewed two clinical leads, both practicing attending hospitalist physicians, and one information systems member. We found three key domains for responding clinicians ( Table 1 ) and three key domains for governance groups, clinical leads, and information systems members ( Table 2 ). We also found three cross-cutting domains related to barriers and one domain related to facilitators of CDS success ( Table 3 ), as well as several recommendations for CDS improvement ( Table 4 ).

**Table 1:** Responding clinician domains.

Domain	Theme	Illustrative interview quotes
Assessing interruptive alert helpfulness	Helpful	Medication alerts, they’re helpful and will change clinical management. – *Responding Clinician 1*
	Unhelpful	The sepsis flag I often disagree with... I don’t think I’ve ever caught new sepsis because of it. – *Responding Clinician 9*
Proportionality	Sensitivity proportional to criticality	For things that are more serious, having false positives is tolerable. – *Responding Clinician 13*
	Interruptiveness proportional to criticality	If it’s a higher safety risk, then I’m fine with it being very prominent and you must respond to it before you can move on. – *Responding Clinician 1*
	Interruptiveness proportional to workflow priorities	If you’re trying to quickly click through patients and throw in orders and you get some alert, you might click through it because you’re not paying as much attention. – *Responding Clinician 13*
Alarm fatigue	Excess false positive interruptive alerts	The best practice advisories keeps coming up, you get alarm fatigue and are kind of ignoring it. – *Responding Clinician 4*

Abbreviation: BPA.

**Table 2:** Governance, clinical lead, and information systems domains.

Domain	Theme	Illustrative interview quotes
Factors that contribute to the high volume and high sensitivity of interruptive alerts	Governance has low tolerance for false-negative interruptive alerts	We argue up against the patient safety folks…there’s always that balance, which I think is heavy toward over alerting is better than under alerting. *– Governance 2*
	Distrust in clinicians	What I hear from my counterparts is they really favor being aggressive because they’re so worried that if we don’t do an interruptive best practice advisories, someone’s going to miss it. We’re not going to do what we were supposed to do... There’s this level of we don’t trust the [end-user] to do the right thing. It’s very frustrating. *– Governance 4*
	Technological solutions for process problems	If you can throw a computer [solution] at something versus a process solution, people always pick the computer solution. Process solutions are hard, operational solutions are hard. *– Clinical Lead 1*
	Reactive to safety events or regulations	If we don’t do an interruptive BPA, someone’s going to miss it. And we’re going to be cited by The Joint Commission, or there’s going to be a patient safety event. *– Governance 4*
Defining interruptive alert success	Different definition of interruptive alert success than clinicians	There’s two levels of performing well in my mind. One is, is the BPA functioning as intended, triggering and firing as it should be? We have an evaluation process to evaluate how the BPA is being utilized. I would say even 20% of the time [accepted], that’s a really good BPA. *– Information Systems 1*
Reluctance to add new interruptive alerts but limited changes to pre-existing interruptive alerts	Reluctance to add new interruptive alerts	It has to be pretty, a very large safety issue or a regulatory thing that would drive us to put a BPA in because we have too many of them. *– Governance 1*
	Limited number of alerts modified or retired	It takes a long time to get things done because [the EHR] over the years has become so bloated with a lot of different rules. – *Clinical Lead 2*

Abbreviations: BPA; EHR, electronic health record.

**Table 3:** Barriers and facilitators to successful clinical decision support.

Domain	Theme	Illustrative interview quotes
**Barriers to successful CDS**		
End-user barriers	Limited knowledge of other CDS types	The other one was a request for [a] best practice advisories for telemetry. There’s so many telemetry orders. The cardiology collaborative suggested a BPA. And we’re like, no, why don’t we instead give guidance at [the] time of ordering low risk telemetry to say this is what the evidence supports. – *Responding Clinician 3*
	Timing of documentation	The timing of [a BPA] is very hard. You’re about to put the discharge order in and I give you a BPA that says five days ago this person had a critically significant result that was not acknowledged. Is that the right place to do this? I don’t think so. But are there better places in advance? Yes, but are those better places noisy? Yes. *– Clinical Lead 1*
	Inaccurate information entered in EHR	Opioids cause itching, and so a long time ago, they’ve been put [in] as an allergy. So, every time an [opioid order] is placed, then that allergy pops up, and you are having to restate that’s not really a real allergy. – *Responding Clinician 10*
Electronic health record technical barriers	Technological limitations on the range of CDS types	Sometimes BPAs are what is at your disposal... there [are] times where you’re like, gosh, I wish instead we could change this other part of [the EHR] that we just don’t have the ability to change. *– Governance 3*
	Multiple ways to do the same task	I can go into [the EHR] and show you at least ten different ways to look at med administrations. The list is infinite in terms of how we obtain the same information. Since you can’t standardize [workflow], I can’t introduce anything into that workflow that will work enough of a time to make it worth it. *– Clinical Lead 1*
Governance-level barriers	Excessive bureaucratic procedure	[The process to change a BPA] can go on several months depending on what it is. The [Office of the Chief Medical Officer] now has an updated policy for IV contrast. Let’s look at the policy and compare them with the BPAs and make sure the BPAs align and are recommending the right thing. But what I just described to you out loud, that’s taken months. *– Information Systems 1*
	Lacking a group solely designated to optimizing CDS	The slowness in which to get things done is reaching out to the right person.... they’d say, I want you to bring it to the [committee] and the next [committee meeting] is not for another month. Sometimes that [committee] may say, well, we think this, but bring it to this other governance group or we should check with [the Office of the Chief Medical Officer]. *– Information Systems 1*
	Limited resources	We’ve probably slacked a bit in the maintenance, updating and reviewing [of BPAs] on the back end. After they’ve been in use for six months, let’s go back and say is this working how we want it to work? Should we leave it on or not? I think creating the time and space to do that would make the [BPAs] in the system more impactful. *– Information Systems 1*
**Facilitators to successful CDS**		
Governance-level facilitators	Organizational governance structure	[We] are the pharmacists, the nurse and the physician [who] are the clinical leads for those domains. And we work with [systems level information systems] when CDS requests come in to help build them. *– Governance 2*
	Silent mode	Before we move the BPAs or any CDS to production environment, we do a lot of silent data collection, usually like a few weeks, sometimes up to a month. *– Clinical Lead 2*

Abbreviations: CDS, clinical decision support; EHR, electronic health record.

**Table 4:** Recommendations for improvement in clinical decision support.

Theme	Illustrative interview quotes
Changing interruptiveness of alerts via flags, changing timing, batching, and alternate forms of CDS	Instead of grinding the work to a halt, the way [best practice advisories] are right now, flagging may be less intrusive. So you’re still faced with the data, but you don’t have to stop everything you’re doing, because it’s flagged. – *Responding Clinician 10*
	In terms of time of day, people who are inpatient [receiving BPAs] in the afternoon is probably better just because that’s when we’re writing notes. – *Responding Clinician 5*
	[If] it’s batched, when you have a minute to go through them, you can resolve them in a lump sum and then move on. They’re less disruptive to the workflow. Not all these BPAs require urgent action. – *Responding Clinician 1*
Making interruptive alerts customizable by user or patient; option to defer or turn off alert	I wish there was a way to set your preferences for BPAs. Like if I were to say, BPA, whenever you calculate the QT, use this Framingham [formula] rather than Bazett’s. And then only show me BPAs that I’ve decided are useful to my clinical practice rather than a pre-populated set that is the same across every user. – *Responding Clinician 15*
	If we’ve acknowledged that someone has a baseline low blood pressure or is tachycardic, are there parameters that we put in our note that [the EHR] would acknowledge and then not pop up a sepsis BPA? – *Responding Clinician 4*
	I wish there was a way to minimize [the BPA] and come back to it later. – *Responding Clinician 15*
Changing interruptive alert design: automating actions, integrating artificial intelligence, changing alert recipient	If [the BPA] could be summarized better and not be huge blocks of text and weird pictures. Maybe it would be more palatable to people. – *Responding Clinician 5*
	[The BPA says] is this a problem? And you say yes and sign, but it’s not forcing you to go back and fix what you just acknowledged was a problem. You have to go back into that previous screen and make those changes. The BPAs that work best [are] when they bring up the order for you. – *Responding Clinician 3*
	A large language model or [artificial intelligence] that can actually interpret what’s going on in the context of the patient’s overall chart would be extremely helpful [in BPA design]. – *Responding Clinician 15*
	Your patient has a central line and doesn’t have chlorhexidine wipes [ordered]. For the love of God, why does the responding clinician have to order chlorhexidine wipes? Can’t that be [a] nursing order? There’s got to be certain things that are not particularly high risk that nurses could do. A lot of [the EHR] the responding clinician has to resolve all of [the BPAs] when it could be a different workflow. – *Responding Clinician 1*
Making interruptive alerts more responsive to medical record, user feedback, and system-wide evaluation	Allergies is another one where it can be useful…oftentimes you get a notification that the patient is listed as being allergic to acetaminophen or [diphenhydramine] when they’ve taken it six times on their most recent hospitalization and no one has updated the allergies. – *Responding Clinician 15*
	If you’ve documented the allergies and you’ve made a decision to order [the medication] anyway... Sometimes we [will] even put a note to the pharmacy [about] our rationale for the override, but the BPA still pops up. If there’s a way for [the EHR] to detect the note to pharmacy to avoid those [BPAs], that would be helpful. – *Responding Clinician 4*
Involve end-users in interruptive alert analysis	The whole life cycle of a BPA and talking to end users is important, but we haven’t made any investments in that. *– Clinical Lead 2*
	No, I don’t think we have a way of users to tell us what’s going right and what’s going wrong beside that comment section, which most of the time is very angry, like any comment section. *– Clinical Lead 1*
	Unfortunately, the way it is designed is the person who requested [the BPA] is not there. So we have to understand what they’re trying to do. Who needs to see it? What is the problem? What is the breakdown? Why exactly do we need it? The whole workflow that you would do in a traditional sort of [quality improvement] process as a process map is not there. *– Clinical Lead 2*

Abbreviations: CDS, clinical decision support; EHR, electronic health record.

### Responding Clinicians Domain 1: Assessment of Interruptive Alert Helpfulness

Clinicians noted that helpful interruptive alerts are “good to know” but often did not change management (Theme 1; Table 1 ). These include pharmaceutical interruptive alerts related to drug–drug interactions and allergies. They also include safety alerts, e.g., about deep vein thrombosis prophylaxis and QTc prolonging medications that could predispose to cardiac arrhythmia. Clinicians also noted interruptive alerts are unhelpful (Theme 2) when they are overly sensitive or technically accurate but not relevant to the patient’s particular situation, resulting in false-positive warnings, i.e., firing when they should not. Frequently, interruptive alerts did not incorporate clinical information in their logic that would have led them to be suppressed. Sepsis was often mentioned as an example of an unhelpful interruptive alert category. Responding clinicians rarely found that sepsis interruptive alerts were accurate or provided new information.

### Responding Clinicians Domain 2: Proportionality

Clinicians frequently noted three different aspects of alert proportionality. The sensitivity of an interruptive alert, i.e., the percentage of alert firing among all cases in which it should fire, ideally should be proportional to the criticality of the alert (Theme 1). For example, responding clinicians felt clinically critical alerts, such as sepsis, should have high sensitivity (i.e., low rate of false negatives), even if it resulted in false positives, but felt less critical alerts should have lower sensitivity. The alert’s interruptiveness should also be proportional to the criticality of the alert (Theme 2), with interruptiveness defined as the degree to which workflow of the end-user in the EHR is interrupted. For example, a clinically critical event should result in an interruptive “hard stop” for the end-user, while less critical events should have less interruptive or noninterruptive alerts. The third aspect is the interruptiveness of the alert relative to end-user’s workflow priorities (Theme 3). Clinicians mentioned if they are busy with a high-priority workflow in the EHR, e.g., writing admission orders, they are more likely to quickly click through interruptive alerts and ignore them.

### Responding Clinicians Domain 3: Alert Fatigue

Clinicians describe alert fatigue (i.e., desensitization to alerts, leading them to be ignored 29 ) due to a high volume of interruptive alerts. Excess false-positive interruptive alerts (Theme 1) result in a loss of trust in interruptive alerts and ultimately end-users overriding most interruptive alerts, regardless of their appropriateness.

### Governance, Clinical Lead, and Information Systems Domain 1: Factors that Contribute to the High Volume and High Sensitivity of Interruptive Alerts

Governance respondents note that quality/safety leadership has low tolerance for false-negative interruptive alerts (Theme 1; Table 2 ). Their biggest concern is that an interruptive alert would not fire when it should and felt the risks of under-alerting outweighed the risks of over-alerting. This attitude reflects inherent distrust in clinicians’ ability to make the best clinical decisions without prompting from alerts or other forms of CDS (Theme 2). Clinical leads and information systems (IS) members expressed that information systems leadership often use interruptive alerts as technological solutions for process problems, driving excess alert use (Theme 3). For example, one participant mentioned interruptive alerts are used to alert users regarding incomplete medication reconciliation. Rather than focusing on ways to assist clinicians and improve medication reconciliation accuracy, e.g., through hiring and training pharmacy technicians, interruptive alerts are used to enforce compliance with a regulatory requirement because they are easier and less expensive to implement than process changes. Lastly, interruptive alerts were often viewed by governance respondents, IS members, and clinical leads as reactive solutions by quality/safety leadership to recent safety events or new systems level regulations (Theme 4), driving up their volume.

### Governance, Clinical Lead, and Information Systems Domain 2: Defining Interruptive Alert Success

Information systems (IS) participants and clinical leads acknowledged that the broader information systems administrative system defines successful interruptive alerts as those that function as designed, i.e., using their internal logic, and provide accurate information to end-users (Theme 1). Interruptive alert success is not defined by acceptance rates, end-user assessment of helpfulness, effect on clinician workflow, effect on clinician’s cognitive load, unintended consequences, or patient outcomes.

### Governance, Clinical Lead, and Information Systems Domain 3: Reluctance to Add New Interruptive Alerts but Limited Ability to Change or Retire Preexisting Alerts

A recurring theme was reluctance by governance groups and the information systems team to add new interruptive alerts (Theme 1). New alert requests from end-users or governance groups are received and reviewed by the information systems group and then considered with collaboration of relevant governance groups and end-user groups. Information systems and governance group interviewees acknowledged an excess of interruptive alerts in the EHR, which is why they carefully deliberate when considering the addition of new alerts. However, the current constrained process of evaluating existing interruptive alerts results in few alert modifications and retirements (Theme 2). The information systems team meets monthly to review existing interruptive alerts with low acceptance rates (usually 1% acceptance or less) and discusses modifying or retiring them. The team brings select interruptive alerts back to the governance groups that approved their initial creation. The information systems team often encounters reluctance from governance groups to modify or retire interruptive alerts, since they are usually the group that recommended their creation. The result is the discontinuation of 0–2 interruptive alerts per month from an existing pool of thousands of interruptive alerts. Participants cited limited staff and time to devote to more rigorous evaluation of existing interruptive alerts or retiring large numbers of them.

### Barriers to Successful CDS Domain 1: End-User Barriers

The information systems group reported that end-users often request interruptive alerts as the primary form of CDS because of limited knowledge about alternative CDS types (Theme 1; Table 3 ). For example, non-urgent noninterruptive alerts about urinary catheter care may present as flags within the chart rather than interruptive alerts that interrupt workflow, but end-users are less aware of this type of decision support and so may not request it. Clinical leads noted that limitations in the timing of EHR documentation also result in difficulty creating accurate CDS (Theme 2). Documentation, e.g., of abnormal vital signs, is often completed retrospectively, making it difficult to design CDS that responds to clinical situations in real time. Finally, clinicians, IS, and governance members noted that end-users entering inaccurate information in the EHR results in ineffective CDS (Theme 3). For example, incorrect allergy documentation makes allergy interruptive alerts inaccurate. Additionally, when patient care team roles are incompletely listed in the EHR, it is difficult to design role- and user-specific CDS, leading to interruptive alerts firing for inappropriate end-users.

### Barriers to Successful CDS Domain 2: EHR Technical Barriers

Governance and clinical lead respondents noted that the EHR presents technological limitations on the range of CDS types that could be supported (Theme 1). For example, it is difficult for the EHR to provide default medication doses and frequencies based on age and renal function rather than reactive and interruptive alerts after orders have been placed. In some cases, less interruptive alerts are technologically possible but more difficult to create than interruptive ones. The EHR also presents limitations in using noninterruptive CDS because of multiple ways to do the same task; there is no way to guarantee the end-user would encounter a specific workflow where noninterruptive CDS could be integrated (Theme 2).

### Barriers to Successful CDS Domain 3: Governance-Level Barriers

Information systems participants identified several governance-level barriers. One barrier is excessive bureaucratic procedures (Theme 1). End-users have limited involvement in the process of developing and reviewing alerts due to the bureaucratic complexities of alert development and retirement workflow. Additionally, the organizational structure lacks an arm solely designated to optimize CDS (Theme 2). Constraints on time, resources, and staff make it difficult to design an ideal CDS review process (Theme 3).

### Facilitators to Successful CDS Domain 1: Governance-Level Facilitators

There were several governance-level facilitators mentioned by participants. One facilitator was the comprehensive existing organizational governance structure around new alert creation (Theme 1). The existing alert development team involves end-users potentially affected by the proposed CDS, which promotes interdisciplinary collaboration with the information systems team. Another facilitator was the practice of testing new alerts in silent mode (Theme 2). Silent mode is when CDS runs as a background process not visible to the end-user. Silent mode is used to gather information about alert firing rate and accuracy, which helps with decisions about whether the alerts should be implemented.

### Recommendations for Improvement

Participants mentioned changing alert interruptiveness with noninterruptive flags, changing the timing of alert delivery or batching noncritical alerts, and using alternate forms of CDS when possible (Theme 1; Table 4 ). Another recommendation was to create customizable CDS (Theme 2). This could include customizable parameters to trigger interruptive alert firing (for specific patients or specific users) and providing options to defer or turn off alerts depending on their type or criticality. They also recommended changing alert design by automating the recommended actions as much as possible, integrating artificial intelligence into alerts, and changing the alert recipient (Theme 3). For example, noncritical alerts related to daytime clinical needs could be presented only to daytime clinicians rather than nighttime clinicians. Participants also recommended changing alerts to be responsive to the medical record, user feedback, and system-wide evaluation (Theme 4). For example, if orders are entered indicating that the end-user is aware of a patient’s hypotension, such as blood culture or lactate orders, the sepsis interruptive alert would not fire. Proposed improvements to the CDS governance system were to streamline the CDS request process and involve end-users in the process of analyzing existing alerts (Theme 5). While the current system promotes interdisciplinary discussion regarding alert creation, it also adds considerable time and complexity to the process. The current process of reviewing alerts for modification/retirement highlights an absence of end-user participation.

## Discussion

At our site, we found many barriers to successful interruptive alerts, grouped by the experiences of responding clinicians or members involved in the systems-level processes around their development and review. We also identified many recommendations for improving CDS, originating from front-line inpatient clinicians, many of which might improve the experience of end-users without compromising (or possibly improving) patient care. Overall, responding clinicians at our site expressed mixed feelings about interruptive alerts, similar to findings of previous studies. 11 They found interruptive alerts’ helpfulness depended on topic category, rates of false positives, and clinical relevance. Responding clinicians mentioned that interruptive alert sensitivity and interruptiveness should be proportional to alert criticality. Responding clinicians expressed a loss of trust in interruptive alerts due to overwhelming interruptive alert volume and high rates of false positives, resulting in responding clinicians overriding most if not all interruptive alerts regardless of helpfulness.

At the systems governance level, participants mentioned barriers to successful interruptive alerts that were a result of different priorities of end-users and systems governance. While end-users wanted high sensitivity interruptive alerts only in highly critical situations, systems governance wanted high sensitivity interruptive alerts under all circumstances, resulting in over-alerting. Overall, systems governance wanted accurate CDS as a means for a systemwide response to internal safety events and external regulations, with little appreciation for how false-positive alerts could negatively affect clinicians and patients.

Our findings can be explained by a variety of factors in today’s EHR landscape. The most significant barriers to successful CDS at our site were limited ways of delivering CDS in the current build of the EHR, limited institutional resources to customize CDS, and fragmentation and limitations of governance. The inherent design of our site’s EHR makes interruptive alerts one of the easiest and least expensive forms of CDS to implement, even though they are known to be disruptive and often ineffective. 5 15 16 Also, unlike custom CDS created during the pre-vendor EHR era, most current alerts at our site were acquired as a bundle from external vendors and are not tiered by criticality. Previous research by Paterno et al showed that by tiering drug–drug interaction alerts in the EHR, acceptance rates of severe and moderate drug–drug interaction alerts increased. 30 This same concept could be applied to all interruptive alerts and potentially result in increased acceptance rates of critical and moderate alerts. While theoretically some of these features are available through customization of the EHR, customization is often not done by individual hospitals after their initial implementation of the EHR. 31 32 Customizing alerts at the institutional level would be ideal but is unfortunately too resource intensive to be a practical and generalizable solution. Most hospitals have limited resources devoted to CDS development and maintenance, and fewer have been able to employ clinical informatics trained clinician-users to provide ongoing feedback. 33 CDS teams may also have limited technical capabilities in creating CDS. Additionally, restraints in governance group capacity may limit further optimization of CDS. Each group involved in CDS governance typically only sees a piece of the health care process and lacks some of the perspectives of others in the organization. For example, patient safety personnel want to make sure a critical safety event does not recur; vendors of alert content want a complete inventory of alerts so they are not faulted for missing anything; end-users want minimal false-positive alerts and interruption to workflow.

Previous research has shown interruptive alert optimization by using strategies of eliciting end-user feedback, reducing alert volume, and reducing inappropriate firing of alerts, similar to the themes identified by participants in our study. 34 35 36 37 Effective governance of CDS has been demonstrated in other institutions, resulting in the transition of alert and reminder content to other more appropriate areas in the EHR, downgrading the severity of alerts (often leading to them no longer firing), 38 39 and assessing the effect of CDS on clinician behavior and patient care. 40 41 Our findings assessing interruptive alert helpfulness and recommendations to change interruptive alert presentation, timing, and targeting were also like those found in previous studies. 35 36 40

A finding not mentioned in previous qualitative CDS research was the contrast between the definition of a successful interruptive alert by end-users and the broader information systems administration. The broader administration defined alert success as alerts functioning as designed and helping the hospital comply with regulations or react to safety events. In contrast, end-users defined alert success as changing clinical management, improving patient outcomes, and having high acceptance rates. Recognizing the incongruent definitions of successful CDS sheds light on one of the drivers of organizational behavior that contributes to ineffective CDS. Another unique contribution our study presents is the explicit concept of assessing CDS on three dimensions of proportionality: alert sensitivity proportional to criticality, alert interruptiveness proportional to criticality, and alert interruptiveness relative to workflow priorities. One unique strength of our study was involving stakeholders from every step in the process of CDS development, governance, and usage. This included involving end-users who were trainees and advanced practice providers, which is especially important within academic teaching hospitals as they often bear the burden of entering medical orders in the EHR and receiving alerts. Few qualitative CDS studies of different stakeholder perspectives have been conducted in the inpatient setting in the modern-day era of vendor-dominated EHRs. 24

A limitation of our study was stakeholders were interviewed at a single hospital within a health system, limiting the generalizability of the results as they may be capturing experiences unique to our single institution. Although we attempted to get a broad, representative group of participants, we were limited by who responded to requests to participate in the study and those respondents may have been more likely to respond because they were either very satisfied or dissatisfied with interruptive alerts. Another limitation of our study is that it only includes end-users in the hospital medicine department (including one hospitalist attending) and not other specialists. Finally, the interview responses of the end-users, mostly residents and APPs, may have been affected by being interviewed by an attending physician.

The findings of our study suggest that attempts should be made to drastically reduce the volume of interruptive alert firing. This would require changing current processes around interruptive alert development, modification, and especially retirement. Interruptive alerts should be tiered by criticality, and less critical interruptive alerts should be presented noninterruptively, allow for batching or suppression, be replaced by other forms of decision support, or eliminated. Critically important but ignored interruptive alerts should be improved. For example, sepsis alerts were almost universally viewed as unhelpful, likely in part due to their high rate of false positives. This points to sepsis alerts being a category ripe for improvement, e.g., by incorporating additional clinical information and clinician actions into the logic. This would provide a framework for modifying other interruptive alerts.

Alerts could be modified by allowing for high sensitivity and interruptiveness for critical alerts and reducing the sensitivity and interruptiveness of nonclinical alerts, e.g., by changing the threshold for firing or presenting the information as an “FYI.” This could also be done by presenting noncritical alerts at less busy times during the end-user’s workflow (e.g., creating a “focus mode” in the EHR and only presenting these alerts when this mode is over). Interruptive alerts could also be modified by who they are presented to, e.g., only the responding clinician from the primary inpatient team. Lastly, interruptive alerts could be made more customizable. For example, a patient with severe systolic heart failure may always have a blood pressure around 90/50—for that patient, the blood pressure threshold for sepsis could be indefinitely changed by a clinician. In other cases, alert logic could be changed at the level of the clinician, for all their patients, based on their level of clinical expertise and familiarity with certain medical conditions.

Ideally, other forms of CDS that are less disruptive to end-users should dominate, including dashboards, just-in-time knowledge bases (info-buttons, web links), automated care pathways, and real-time recommendations. For example, CDS could recommend the dose and frequency of medications based on patients’ age and renal function at the time of ordering, rather than firing an interruptive alert after the order has been placed. 42 We should note that interruptive alerts can also provide real-time recommendations, which would make them more useful than those that do not, but they still carry the risk of alert fatigue.

Lastly, in all cases, the effects of CDS on clinician workflow, cognitive load, and patient outcomes need to be measured. It is insufficient to evaluate CDS based on whether their logic fires as intended and by acceptance rates alone.

## Conclusion

While interruptive alerts were initially developed as a tool to enhance patient care, they have resulted in clinician alert fatigue and workflow disruption due to their high volume and false-positive rate. Differences in priorities of systems governance and end-users as well as EHR limitations contribute to the dysfunctionality of interruptive alerts. End-users wanted alerts that were customizable, less disruptive, and lower in volume. Ideal future CDS would be centered on the principles of Osheroff’s Five Rights of CDS and anticipate end-user needs to enhance clinician efficiency and improve clinical outcomes. 27 Devoting more resources to alert development, maintenance, and retirement with end-user involvement would be an important step toward realizing the full potential of CDS.

**Clinical Relevance Statement**

The implications of this study are that end-users wanted clinical decision support (CDS) that was relevant, targeted, less interruptive, and customizable. Optimizing interruptive CDS alerts, or “pop-ups,” would specifically involve reducing false-positive alerts, reducing alert interruptiveness when appropriate, and involving end-users in alert development, maintenance, and retirement. CDS needs to be revolutionized to fulfill its original purpose of enhancing clinician efficiency and improving patient care.

**Multiple-Choice Questions**

1. Which factor contributed to end-users ignoring interruptive alerts in the study?
  - Insufficient training on alert usage
  - High rates of false-positive interruptive alerts
  - High tolerance for false-negative interruptive alerts by governance groups
  - Batching alert delivery
  **Correct Answer** : The correct answer is option b. End-users commonly reported ignoring interruptive alerts due to the high rates of false-positive alerts and high alert volume.
2. Why are interruptive alerts commonly used as a form of CDS?
  - Interruptive alerts are the preferred format for CDS by end-users
  - Interruptive alerts have been shown to be the most effective form of CDS
  - Interruptive alerts are easier to implement compared with many other forms of CDS
  - Interruptive alerts have the highest acceptance rates among end-users
  **Correct Answer** : The correct answer is option c. Interruptive alerts are often used as a form of CDS because they are easy to implement compared with many other forms of CDS, especially in modern vendor-dominant EHRs commonly used in the inpatient setting.

## Supplementary Material

Ergänzendes Material Supplementary Material
