# Supplementary material for: Qualitative Study of Inpatient Hospital Medicine Interruptive Alerts and Clinical Decision Support
Source: Appl Clin Inform. 2026 Oct 12;17(4):889–98. doi: 10.1055/a-2955-9555 (PMC13657796; doi:10.1055/a-2955-9555)
Supplement: Supplementary file 1 [file 10-1055-a-2955-9555_29782387.pdf]

Supplementary Appendix

1. Semi-structured interview guides by stakeholder group

a. Semi-structured interview guide for responding clinicians (RCs):

One way electronic medical records aid in clinical decision making is through best practice advisories (BPAs), sometimes known as pop-ups, which are prompts that alert the clinician to pertinent information when a patient's chart is opened or orders are placed. Inpatient clinicians receive a high volume of BPAs. We want to understand your opinions of Epic BPAs and the ideal method of delivering clinical decision support at Brigham and Women's Hospital.

1. Before I get into further questions, I would like to take a moment to address any questions you may have about BPAs and how they work.
2. How do you feel about BPAs?
   a. [Prompt] How do you perceive your colleagues feel about them?
3. Tell me about your interactions with BPAs.
   a. [Prompt] When do you typically receive them?
   b. [Prompt] How many do you get in a day?
   c. [Prompt] How often do you ignore them?
   d. [Prompt] Why do you ignore them?
4. When you read them, how often do they provide useful information?
   a. [Prompt] Why is the information useful or not useful?
5. How often do you accept their suggestions?
   a. [Prompt] Why do you or don't you accept the suggestions?

6. Tell me about the last time you were taking care of a patient and a BPA fired that affected the way you took care of that patient.

   a. [Prompt] And if not, why?

   b. [Prompt] If you have seen BPAs affect patient care positively, describe the positive effects.

   c. [Prompt] If you have seen BPAs affect patient care negatively, describe the negative effects.

7. Could you give examples of types of BPAs that are helpful or unhelpful?

   a. [Prompt] For example, sepsis BPAs, drug-drug interaction BPAs, foley BPAs, DVT BPAs, qtc bpa, fall risk BPA, order BCx BPAs, code status BPA, start antibx BPAs, allergy BPAs?

8. How are BPAs not helpful?

   a. [Prompt] How could they be improved?

   b. [Prompt] Timing, interruptive, volume, targeting the right person, quality of the advice, receiving at admission vs. discharge, other?

9. In approximately what percentage of BPAs would you say you accept the suggestion? What percent do you override?

10. How do you feel about false positive BPA's firing?

    a. [Prompt] When do you feel like it's not a big deal and when is it annoying? Is it affected by timing, patient load?

    b. [Prompt] What types of BPAs would you be okay with false positive BPAs firing?

11. Are BPAs compatible with your current workflow?

a. [Prompt] Why? Why not?

b. [Prompt] How could BPAs be better adapted to your workflow?

12. Tell me about any workarounds or improvisations that you use with BPAs.

13. What might be another helpful way to communicate some of the information delivered in a BPA?

14. Is there anything we didn't talk about that you would like to share or think is important to know?

b. Semi-structured interview guide for clinical leads:

One way electronic medical records aid in clinical decision making is through best practice advisories (BPAs), sometimes known as pop-ups, which are prompts that alert the clinician to pertinent information when a patient's chart is opened or orders are placed. Inpatient clinicians receive a high volume of BPAs. We want to understand your opinions of Epic BPAs and the ideal method of delivering clinical decision support at Brigham and Women's Hospital.

1. Before I get into further questions, I would like to take a moment to address any questions you may have about BPAs and how they work.

2. How do you feel about BPAs?

   a. [Prompt] How do you perceive your colleagues feel about them?

3. Tell me about your process of receiving a request for a BPA.

   a. [Prompt] Who requests them?

   b. [Prompt] How many requests do you get in a week or month?

   c. [Prompt] How often do they result in a BPA versus a different form of CDS?

4. Do BPAs affect patient care?

a. [Prompt] If so, how?

b. [Prompt] And if not, why?

c. [Prompt] If you have seen BPAs affect patient care positively, describe the positive effects.

d. [Prompt] If you have seen BPAs affect patient care negatively, describe the negative effects.

5. How and which BPAs are helpful?

a. [Prompt] For example, sepsis BPAs, drug-drug interaction BPAs, allergy BPAs?

6. How are BPAs not helpful?

a. [Prompt] How could they be improved?

b. [Prompt] Timing, interruptive, volume, targeting the right person, quality of the advice, other?

7. Should BPAs be changed? Why or why not?

a. [Prompt] Why or why not?

b. [Prompt] How do you think BPAs compare to other forms of CDS? Advantages vs disadvantages?

8. Approximately what percentage of your week is spent on work supporting BPAs?

9. Is there anything we didn't talk about that you would like to share or think is important to know?

c. Semi-structured interview guide for governance groups:

One way electronic medical records aid in clinical decision making is through best practice advisories (BPAs), sometimes known as pop-ups, which are prompts that alert the clinician to pertinent information when a patient's chart is opened or orders are placed. Inpatient clinicians receive a high volume of BPAs. We want to understand your opinions of Epic BPAs and the ideal method of delivering clinical decision support at Brigham and Women's Hospital.

1. Before I get into further questions, I would like to take a moment to address any questions you may have about BPAs and how they work.
2. Tell me about your thoughts about BPAs from a governance group perspective? How about from a clinician perspective?
3. Can you tell me about your involvement in creating and approving a new BPA?
    a. [Prompt] What factors go into the decision to create and approve a new BPA?
4. Can you tell me about your involvement in modifying or retiring a BPA?
    a. [Prompt] What factors go into the decision to modify or retire a BPA?
    b. [Prompt] Can you give an example of a BPA that was modified and/or retired? Why was it modified and/or retired?
    c. [Prompt] Can you give an example of a BPA where the decision was made not to modify and/or retire it despite the suggestion to do so? Why was that decision made?
5. How you feel about BPAs?
6. Do you participate in direct care for patients?
7. Do you interact with BPAs on a consistent and regular basis?

a. [Prompt] If so, how many do you get in a day?

i. [Prompt] How often do you ignore them? Why do you ignore them?

ii. [Prompt] When you read them, how often do they provide useful information? Why is the information useful or not useful?

iii. [Prompt] How often do you accept their suggestions? Why do you or don't you accept the suggestions?

b. [Prompt] If not, describe the experiences and interactions you have had with BPAs.

8. How do you perceive responding clinicians (clinicians who place orders and therefore receive BPAs on a consistent and regular basis) feel about them?

9. How are BPAs helpful? How are they not helpful?

a. [Prompt] How could they be improved?

10. Do you have any suggestions about how they could be better?

a. [Prompt] Content, volume, timing, right recipient, non-interruptive

11. Can you think of other ways to deliver decision support other than BPAs?

12. Is there anything we didn't talk about that you would like to share or think is important to know?

d. Semi-structured interview guide for information system (IS) members:

One way electronic medical records aid in clinical decision making is through best practice advisories (BPAs), sometimes known as pop-ups, which are prompts that alert the clinician to pertinent information when a patient's chart is opened or orders are placed. Inpatient clinicians receive a high volume of BPAs. We want to understand your opinions of Epic

BPAs and the ideal method of delivering clinical decision support at Brigham and Women's Hospital.

1. Before I get into further questions, I would like to take a moment to address any questions you may have about BPAs and how they work.
2. Can you tell me about the last time you created and implemented a new BPA? What was that process?
3. Can you tell me about the process and your involvement in retiring or modifying BPAs?
4. What are the barriers to retiring or modifying poorly performing BPAs?
5. Can you tell me what you like or don't like about working with BPAs?
    a. [Prompt] How do you think the process could be improved?
6. Do you have a sense of what the clinicians using BPAs think of them and what their perceptions are?
7. Do BPAs affect patient care?
8. How are BPAs helpful or not helpful?
    a. [Prompt] Do you have any suggestions about how they could be better?
    b. [Prompt] Content, volume, timing, right recipient, non-interruptive
    c. [Prompt] Should BPAs be changed? Why or why not?
9. What other forms of CDS are effective alternative to BPAs?
    a. [Prompt] Do you think some BPAs should be turned into other forms of decision support?
10. Approximately what percentage of your week is spent on work supporting BPAs?

11. Is there anything we didn't talk about that you would like to share or think is important to know?

2. **Recruitment material by stakeholder group**

   a. **Recruitment verbal announcement for RCs:** We are recruiting participants who have had at least 3 months of consistent, regular BWH Epic use for a study to understand the perspectives of end users on best practice advisories (BPAs), sometimes known as Epic pop-ups. These are prompts that alert the clinician to pertinent information when a patient's chart is opened or orders are placed. We want to understand your opinions of Epic BPA's and the ideal method of delivering clinical decision support at Brigham and Women's Hospital.

   b. **Email for recruitment of RCs:** We are recruiting participants who have had at least 3 months of consistent, regular BWH Epic use for a study to understand the perspectives of end users on best practice advisories (BPAs), sometimes known as Epic pop-ups. These are prompts that alert the clinician to pertinent information when a patient's chart is opened or orders are placed. We want to understand your opinions of Epic BPAs and the ideal method of delivering clinical decision support at Brigham and Women's Hospital. If interested in participating in an interview about your thoughts on BPAs, contact akhan56@bwh.Harvard.edu.

   c. **Email for recruitment of governance group members:** We are recruiting participants who have had experience in the oversight process of Epic alerts for a study to understand the perspectives of governance groups on practice advisories (BPAs), sometimes known as Epic pop-ups. These are prompts that alert the clinician to pertinent information when a patient's chart is opened or orders are

placed. We want to understand your opinions of Epic BPAs and the ideal method of delivering clinical decision support at Brigham and Women's Hospital. If interested in participating in an interview about your thoughts on BPAs, contact akhan56@bwh.Harvard.edu.

d. **Email for recruitment of IS members:** We are recruiting participants who have had experience in the process of analyzing Epic alerts for a study to understand the perspectives of IS team members on practice advisories (BPAs), sometimes known as Epic pop-ups. These are prompts that alert the clinician to pertinent information when a patient's chart is opened or orders are placed. We want to understand your opinions of Epic BPAs and the ideal method of delivering clinical decision support at Brigham and Women's Hospital. If interested in participating in an interview about your thoughts on BPAs, contact akhan56@bwh.Harvard.edu.

e. **Email for recruitment of clinical leads**: We are recruiting participants who have had experience in the process of creating Epic alerts for a study to understand the perspectives of Clinical Leads on best practice advisories (BPAs), sometimes known as Epic pop-ups. These are prompts that alert the clinician to pertinent information when a patient's chart is opened or orders are placed. We want to understand your opinions of Epic BPAs and the ideal method of delivering clinical decision support at Brigham and Women's Hospital. If interested in participating in an interview about your thoughts on BPAs, contact akhan56@bwh.Harvard.edu.

3. **Supplementary Table S1 Factors influencing interruptive alert governance throughout the alert life cycle**

**Governance Stage**	**Influencing Factors**
Initiation of new alert request	Patient safety event, regulatory or accreditation requirements, new clinical guidelines, performance on quality metrics, change in institutional workflow, payor requirements, healthcare system priorities.
Decision to create a new alert	Medical-legal risk, anticipated change in end-user behavior, clinical value, non-interruptive alert alternatives, IS resources, concerns for alert fatigue, frequency of alert firing.
Re-evaluation of existing alert	Feedback, alert override and acceptance rate, frequency of alert firing, change in institutional workflow, change in electronic health record capabilities, regulatory changes, clinical guidelines.
